# Research on the Cooperative Governance Path of Multiple Stakeholders in Doctor–Patient Disputes under the Environment of Information Asymmetry

**DOI:** 10.3390/ijerph20021597

**Published:** 2023-01-16

**Authors:** Mao-Min Jiang, Zheng-Yu Wu, Ai-Xian Tu

**Affiliations:** 1 School of Public Affairs, Xiamen University, Xiamen 361005, China; 2 School of Management, Hainan Medical University, Haikou 571199, China

**Keywords:** doctor–patient disputes, dispute governance, grounded theory, multiple co-governance

## Abstract

The number of doctor–patient conflicts and disputes in China has been increasing recently. In order to solve the current social problems of the tense doctor–patient relationship and frequent medical disputes, this article, based on grounded theory, uses qualitative analysis software to conduct grounded coding on 622 cases of doctor–patient disputes randomly selected by stratification. After successively adopting open, axial, and selective coding, the relationship structure between the causes and development of medical disputes is summarized. Furthermore, this relationship structure can be used to analyze further and discuss the causes of doctor–patient disputes from the perspective of multiple co-governance and the governance framework of doctor–patient disputes. Finally, it reminds us of the need to standardize government’s regulatory responsibilities, promote the equal distribution of medical resources, strengthen the communication awareness between doctors and patients, reduce the information asymmetry between doctors and patients, and build a preventive mechanism-oriented multi-subject collaborative governance path to promote the relationship between doctors and patients in China. We provide relevant countermeasures and suggestions for harmonious development and the smooth resolution of doctor–patient conflicts.

## 1. Introduction

The Chinese government, which has permanently attached great importance to building a harmonious relationship between doctors and patients, put forward the core value concept of “patient-centered” care, advocating the public welfare and humanization of medical services, and has promoted the construction of a new type of doctor–patient relationship; the government has also implemented a people’s mediation mechanism, dispute prevention mechanism and other measures to create a positive and healthy relationship between doctors and patients. However, the relationship between doctors and patients in China has not improved fundamentally, and the number of judicial cases between doctors and patients is also increasing yearly [1]. The contradiction between doctors and patients has become a hot and challenging issue in constructing a harmonious society. Doctor–patient disputes result from the evolution of tense relationships. Usually, they refer to disputes and conflicts between doctors and patients due to medical services or other factors other than medical services. Doctor–patient disputes will not only directly lead to the outbreak of conflicts between doctors and patients but also lead to the two sides confronting each other head-on; it will also reduce the level of trust between doctors and patients, bring the diagnosis and treatment process to a deadlock, and affect the quality and level of diagnosis and treatment [2]. In a new era and stage for the implementation of an epidemic prevention and control strategy and the implementation of the Healthy China strategy, the governance of doctor–patient disputes reflects the distinctive characteristics of the times, a clear problem orientation, and vital innovation needs [3]. In the post-epidemic era, how to scientifically and effectively govern doctor–patient disputes has aroused extensive discussions in all walks of life [4]. The research on doctor–patient dispute governance is not only a direct response to building a harmonious doctor–patient relationship but also a requirement of the times to promote the implementation of the “Healthy China” strategy and the construction of a harmonious society [5]. Based on the theoretical framework of collaborative governance and from the perspective of collaborative participation of multiple subjects, this study uses grounded theory to study and discuss the path of medical dispute governance, providing a theoretical reference for government departments or medical institutions to prevent and resolve medical disputes correctly.

Scholars at home and abroad have conducted much research on doctor–patient disputes. After sorting, it is not difficult to find that the current research mainly focuses on the cause analysis and countermeasures of doctor–patient disputes. In terms of the causes of doctor–patient disputes, Parsons put forward the theory of the patient’s role, in 1951, pointing out that there is a significant asymmetry between doctors and patients, and misunderstandings are prone to lead to opposition between doctors and patients [6]; in 1956, Hollender and Szasz also proposed a doctor–patient relationship consisting of a “mutual participation model, guided cooperation model, and active-passive model,” pointing out that because doctors fail to use different diagnosis and treatment models according to their conditions during diagnosis and treatment, it has caused tension between doctors and patients [7,8]. However, Allman believes that communication between doctors and patients plays a crucial role. The deep cause of doctor–patient disputes stems from the lack of in-depth communication and communication [9]. When Chinese scholars discuss the causes of doctor–patient disputes, they attribute it to the lack of trust between doctors and patients, information asymmetry, and uneven distribution of medical resources. For example, Li pointed out that medical staff believed that patients who came to seek medical treatment perceived doctors as having a “condescending” mentality, and that they ignored listening to patient’s complaints and questions [10]; Qi said that with the professionalization of doctors and the industrialization of medical institutions, doctors and the trust between patients gradually dissipated, which eventually led to the occurrence of doctor–patient disputes [3]; Shao and Hu believed that information asymmetry between doctors and patients led to wrong choices, moral hazard and one-sided judgments, which made patients feel the dissatisfaction of doctors accumulates and promotes the transformation of the doctor–patient relationship from dissatisfaction to disputes [11]; Wu and Meng believed that the imbalance of medical resources made the medical needs of patients unable to be met from the perspective of social and economic development, and this caused doctor–patient disputes [12].

Scholars at home and abroad have also conducted in-depth discussions on the path and countermeasures of doctor–patient disputes. For example, Chen Zheng and Peng Hua proposed that hospitals should make full use of the information system’s key monitoring role in medical safety indicators and establish a risk sentinel early warning mechanism to deal with potential disputes [13]; Gerds and Lerner pointed out that artificial intelligence should be used to reduce medical risks The advantages of prediction and management are to quantify medical risk early warning indicators and establish a risk early warning system. Hospitals should closely prevent and control potential risk factors with higher risk levels to make effective interventions as soon as possible when doctor–patient disputes occur [14,15]; Rubin believes that hospitals should focus on the quality of medical care, play an active role in the risk management of evidence-based medicine in the process of diagnosis and treatment, and curb the emergence of doctor–patient conflicts from the source [16]; while Zhang, Hu, Tao, etc. believe that the government should play a role in governance. They pointed out that the government should scientifically design the framework of laws and regulations for doctor–patient disputes in China, clarify critical points such as risk prevention and patient-centeredness [17,18,19], and should give full play to the role of third-party mediation mechanisms in resolving doctor–patient disputes [20,21].

The existing research literature shows that although domestic and foreign scholars have conducted rich research on the causes and solutions of doctor–patient disputes, the current research still presents fragmentation and local characteristics and fails to respond from a systematic and global perspective. The governance theory emphasizes “multiple subject participation, clear responsibilities, and fair and reasonable goals”. Grounded theory is a qualitative research method that uses systematic procedures to summarize and guide grounded theories according to the development direction of social problems. Therefore, from the perspective of multi-subject collaborative governance, this paper uses grounded theory to explore the path framework of doctor–patient dispute governance under multi-stakeholders.

## 2. Methodology

We used grounded theory to conduct coding analysis on the research text data. We used NVIVO software to perform open, axial, and selective coding during the coding process. During the coding process, the Delphi method was used to obtain the opinions of external experts for some objectionable content. Open coding is a process of breaking up data, assigning concepts to the data, and then recombining the data in a new way through the careful study of phenomena, that is, the process of analyzing, inspecting, conceptualizing, summarizing, and comparing data. The purpose of open coding is to discover conceptual categories from the data and name the categories to determine the attributes and dimensions of the categories. The code is saturated. In the process of open coding, a dozen or even hundreds of concepts will be generated, so researchers must classify similar concepts, but the concept of categories in this stage is temporary and may be changed at any time due to situations where a discovery necessitates modification of the genus [22]. Axial coding is a complex process of linking similar codings together through continuous comparison, and its main task is to select and construct the content of the main categories [23] and connect the main concept categories with the secondary concept categories to find the underlying logical connections between the various genera eventually form tighter categories [24]. The purpose of selective coding is to further systematically deal with the relationships between categories. The main work is to summarize the “Core category” through integration and to condense and explain all phenomena in the form of a “Story Line” [25]. The core category is the keywords obtained after condensing all the analysis results, and the condensed keywords are enough to explain the connotation of the fundamental research.

The data used in the analysis of this article come from the 2020 medical dispute cases of China Judgment Documents Network, and the judicial cases of doctor–patient disputes are selected as the analysis data of this article. The cause, mediation, and result of the event are representative, fair, and traceable, so we can better understand the evolution and development of doctor–patient disputes; Secondly, judicial cases have recorded the causes and consequences of doctor–patient disputes in detail, and the records are completely authentic and reliable, and the quality of the materials is relatively high. In the China Judgment Documents Network, we searched by setting the case type as a civil case, the cause of the case as medical liability dispute, the trial procedure as a civil first instance, and the date of judgment as after 2020. A total of 15,210 case texts were retrieved, including 13,533 cases of doctor–patient conflicts in public hospitals. After eliminating 7316 invalid texts, such as duplicate case names, undisclosed content, and civil rulings, 6217 samples remained. The sample size of the analysis is between 30–500 [26,27], so we used IEAD software to conduct stratified sampling according to the ratio of 1/10, to try to include every province in China in the research scope, and, finally, screen out 622 case texts. The total number and sampling quantity of doctor–patient disputes in various provinces of China are shown in Table 1.

## 3. Results

### 3.1. Open Coding

In this paper, after reading and analyzing the text data word by word and carrying out initial conceptualization, 1306 original sentences and corresponding initial concepts were finally obtained, the initial concept of frequency three and above. Table 2 shows the obtained initial concepts and categories. In order to save space, three to four original sentences for each category are excerpted in Table 2.

### 3.2. Axial Coding

This paper summarizes six principal categories according to the central axis coding through our repeated derivation and induction. The meaning of each main category and its corresponding sub-categories is shown in Table 3.

### 3.3. Selective Coding

Through the analytical step of selective coding, researchers developed faction types on which to build a theory. In this paper, 17 sub-categories were identified, such as “improper use of drugs, improper operation, insufficient risk notification, and lack of trust in diagnosis and treatment,” as well as “doctor’s fault in diagnosis and treatment, improper medical behavior, lack of hospital management, lack of government supervision, subjective dissatisfaction of patients, and objective factors. The investigation of six main categories, such as “factor constraints”, determined the story line of multi-stakeholders’ collaborative participation in governance: the government, hospitals, doctors, patients, and other stakeholders have some improper behaviors in medical activities, and these behaviors directly or indirectly affect doctor–patient disputes. The emergence and development of doctor–patient disputes have an impact, and to avoid doctor–patient disputes requires multiple subjects to manage these direct or indirect influencing factors jointly.

## 4. Discussions

According to the typical relationship structure between the main categories in Table 4, the internal formation mechanism of doctor–patient disputes from the perspective of multiple subjects can be drawn, that is, lack of government supervision, inadequate hospital management, improper medical and nursing behavior, and doctor’s fault in diagnosis and treatment. The mutual influence and effect of other factors directly or indirectly lead to the occurrence and development of doctor–patient disputes.

### 4.1. In Order to Obtain the Most Incredible Economic Benefits, Medical Institutions Lay Hidden Dangers of Disputes

Under China’s current medical and health system, government departments generally adopt the method of formulating rules to regulate independently operated medical institutions in the supervision of medical institutions. Usually, government departments do not directly supervise the daily operation of medical institutions [28]. Due to the large number of medical institutions in China, the operation of the regulatory mechanism requires a large workforce, material resources, and financial resources [29], and there are no low regulatory costs. The income and development of medical institutions bring “income generation” to government departments. Therefore, some government departments tend to relax or even downplay their regulatory responsibilities for medical institutions out of self-interest considerations. As a unique service organization, medical institutions ensure their essential operation and development needs. Taking measures to obtain profits has become the primary goal that medical institution managers must implement. Therefore, they will adopt non-practicing medical services and use inferior medical equipment. These behaviors will adversely affect the service level of medical institutions and will also lay hidden dangers for future doctor–patient disputes. At the same time, to obtain the most significant economic benefits, doctors will also prescribe more medicines, more expensive medicines, and more checklists, which not only aggravates the dissatisfaction of patients with medical staff but also catalyzes the emergence of doctor–patient disputes and development.

### 4.2. Lack of Medical Resources Increases the Probability of Doctor–Patient Disputes

Disease diagnosis and treatment depend on high-quality medical technology and advanced medical equipment [30]. When seeing a doctor, medical equipment and staff, as critical medical resources, directly affect the patient’s diagnosis and treatment. In an example of a dispute case, the hospital “did not have the necessary rescue equipment and rescue technology, resulting in the death of the patient in the hospital because he could not receive proper treatment after suffocation,” and “the level of medical technology at that time could not be adjusted to the fetal stage. Diagnosis of hereditary diseases”, leading to patients’ high dissatisfaction with the results and quality of diagnosis and treatment, which eventually led to disputes and conflicts between the two parties. Currently, the total medical resources in China are insufficient and unevenly distributed between regions. There are differences in medical resources and technologies between urban and rural hospitals and between public and private hospitals. The medical service system and the people’s medical treatment needs differ. There will inevitably be an increase in the probability of doctor–patient disputes.

### 4.3. Insufficient Communication Leads to Disputes

Communication is a bridge of trust between people and a way of interaction and collision between thoughts and emotions. Good communication is conducive to the construction of trust and friendly interpersonal relationships [31]. In the process of seeing a doctor, the patient’s anxiety and expectation of disease treatment make him subjectively eager to get communication and feedback from the doctor to relieve his inner anxiety and panic. However, in the communication process between doctors and patients, doctors cannot fully understand the emotional needs of patients, and they also lack psychological empathy for patients. In most cases, doctors only pay attention to understanding the patient’s physiological condition and specific symptoms in the process of diagnosis and treatment, ignoring the information communication and emotional exchange with the patient. Just as in the case, the medical staff “did not notice the abnormal behavior of the patient Li, and the mood swings were obvious,” “the communication with the patient about the significance of cerebral angiography was insufficient, and the communication between the doctor and the patient was insufficient,” there was no communication between the doctor and the patient. With direct, effective, and sufficient communication and exchanges, patients can understand the doctor’s diagnosis, treatment ideas, and treatment plans. Doctors also cannot feel the patient’s psychological changes and inner dissatisfaction. The lack of communication between the two parties has resulted in a lack of information exchange and emotional connection. Obstacles catalyze the emergence of conflicts between doctors and patients and eventually lead to disputes.

### 4.4. Moral Hazard Promotes the Development of Disputes

In medical and health services, hospitals and medical staff have absolute information advantages, which are not only reflected in the grasp of medical knowledge but also in the selection of treatment plans and drugs. It is because doctors have absolute information advantages. Doctors are prone to moral risks such as insufficient respect for the right to informed consent and inducing demands during the treatment process, which leads to misunderstandings between doctors and patients and aggravates the development of disputes [32]. For example, “doctors did not inform patients and their families about the risks and alternatives of brain damage from surgery” before surgery, and “the doctor communicated insufficiently and inadequately with patients on the significance of cerebral angiography”. However, these measures will not directly affect the diagnosis and treatment results. However, it violates favorable principles from the medical ethics perspective, which will inevitably deepen the misunderstanding between doctors and patients and then quickly escalate disputes between doctors and patients. In addition, due to the interference of factors such as economic benefits and imperfect supervision mechanisms, some doctors use the advantages of information to induce demand for patients and provide patients with medical services that do not meet the needs of diagnosis and treatment. Trust will plummet, and the doctor–patient relationship will undoubtedly deteriorate faster.

### 4.5. Medical Errors Stimulate the Outbreak of Disputes

In the disease diagnosis and treatment process, patients and their families often have higher requirements and expectations for the results of diagnosis and treatment. However, some medical staff may have misdiagnoses, improper clinical operation, and other faulty diagnosis and treatment behaviors due to a lack of professional skills [33]. For example, in one of the cases, the medical staff “diagnosed acute pharyngitis as acute pharyngitis and treated it with antibiotics in a hanging bottle when the test results were abnormal,” and “the wrong implementation of hysterectomy caused the plaintiff’s cervical cancer gland”; it may also be due to objective technical resource conditions. Restrictions lead to mistakes in diagnosis and treatment. For example, in this case, “the hospital did not need rescue equipment, and the patient died in the hospital because he could not get the proper treatment after suffocation”. However, medical errors, no matter the cause, will seriously lower the expectations of patients and their families and lead to dissatisfaction among patients. In addition, patients have an insufficient accumulation of medical professional knowledge or lack of communication, and the dissatisfaction and grievance of patients quickly accumulate. The doctor–patient relationship deteriorates rapidly, resulting in doctor–patient disputes. In addition, some patients and their families are emotionally unable to accept all negative treatment results and have little tolerance for medical errors within the normal range. Psychologically provoked incidents make the contradictions and conflicts between doctors and patients more intense.

## 5. Recommendations

Participation in the whole process is the basic premise of the collaborative governance of multiple subjects [34]. The governance of doctor–patient disputes involves multiple stages and is long-term. Only by participating in the process can we ensure a comprehensive grasp of doctor–patient dispute information and avoid further conflicts caused by information asymmetry [35]. In addition, communication is the information link for the collaborative governance of multiple subjects. In the process of doctor–patient dispute governance, the communication and exchange of multiple subjects on dispute governance measures can not only enrich the information and requirements related to dispute governance, improve the rationality and scientificity of dispute governance measures, but also better help each participant. Understand and accept governance measures [36]. Moreover, the governance goal is a highly concentrated governance direction and concept, representing doctor–patient dispute governance’s value orientation. Because of the particularity of doctor–patient disputes and the bottom-line thinking of people first and safeguarding the people’s fundamental interests, protecting the legitimate rights and interests of both doctors and patients is undoubtedly the fundamental goal of doctor–patient dispute governance [37]. Therefore, multi-subject collaborative governance should not only realize the disadvantaged position and low risk-tolerance of patients in the medical business but also pay attention to the interests of patients in the governance process and ensure the participation and voice of patients. In most cases, doctors have no intention to create doctor–patient disputes subjectively and intentionally. Finally, promoting the healthy development of the medical industry is the inherent goal of managing doctor–patient disputes. It is necessary to follow the natural law of disease outcomes and to accommodate seemingly disadvantaged groups only a little. The governance measures need to consider realistic factors objectively and fairly.

### 5.1. Standardize the Responsibilities of Government Supervision and Improve the Internal Management of Hospitals

Clarifying the government’s regulatory responsibilities and standardizing the government’s regulatory behavior is a crucial way to urge government departments to improve the service efficiency of medical institutions. First of all, it is necessary to promptly stop hospitals’ improper behavior through the government’s accountability mechanism. At the same time, it is necessary to promote further the formation of the “separation of management and management” pattern from the practical level so that the regulators and the regulated have no common interests and promote the supervision of medical institutions by the regulatory authorities to normalization, standardization, and institutionalization, development in the direction of refinement. Regarding the internal management problems of the hospital, public hospitals should further promote the reform of the hospital’s internal personnel management, salary system, performance appraisal, etc., so that the hospital will gradually change from “pursuing benefits” to “returning to public welfare,” and by solving the “income worries” of medical staff, “To avoid their intrinsic motivation to pursue economic interests and guide them to have the correct value orientation. As for private hospitals, they should improve their internal supervision and control mechanism, give full play to the supervisory role of third-party organizations such as news media and industry associations, improve the self-discipline of medical staff in private hospitals, strengthen publicity and education for medical staff, and cultivate a sense of identity, belonging, and honor to one’s profession, correcting career motivations, and truly “saving the dying and healing the wounded”.

### 5.2. Balance the Distribution of Medical Resources and Deepen the Reform of the Diagnosis and Treatment System

Given the current situation of uneven distribution of medical resources, the government should give full play to the function of macro-control, starting from the aspects of hardware facilities, medical staff, science and technology, and encouraging learning to promote the continuous reduction of medical disparities between urban and rural areas and between regions. For example, for areas with relatively weak medical resources, the government should make full use of financial transfer, policy assistance, etc., increase capital investment, and improve the hardware facilities of local medical institutions, so that they will not be unable to meet the needs of patients due to backward equipment and defective equipment. With respect to the demand for medical treatment, medical staff can be selected from the tertiary hospitals with relatively concentrated medical resources to assist in areas with relatively weak medical resources, or excellent medical staff can be selected from the areas with relatively weak medical resources to go to the tertiary hospitals for further training, improving the overall level of local medical staff. In the face of insufficient resources, the development and improvement of the hierarchical diagnosis and treatment system should be promoted. By accelerating the improvement of the medical pattern of “first diagnosis at the grassroots level, two-way referral, separation of acute and chronic diseases, and linkage between upper and lower levels,” the situation of tight medical resources can be effectively solved. For example, the government should concentrate on building grassroots medical and health institutions, provide preferential policies to attract investment, and accelerate the development of grassroots health services; optimize and redistribute existing medical and health resources, merge medical institutions with overlapping functions and close geographical locations, and large hospitals’ partnerships with grassroots hospitals can be established to realize the sharing of medical resources. The medical service system can be further optimized through models such as medical alliances and alliances. The effect of valuable medical resources can be amplified so that people can seek medical treatment in time at their doorsteps.

### 5.3. Strengthen the Awareness of Doctor–Patient Communication and Improve the Art of Communication between Diagnosis and Treatment

Doctor–patient communication is an art, a required professional course for medical staff, and a meaningful way to ease disputes between doctors and patients. For the current domestic situation, strengthening the communication between doctors and patients must start from two aspects: changing the service concept of medical staff and improving communication skills. For example, in the doctor’s inpatient training stage, the concept of doctor–patient communication is taken as one of the critical points of the training. The training content can cover the service concept, the standard of words and deeds of the service process, the importance of doctor–patient communication, the emotional intelligence education of medical staff, and the development of doctor–patient communication. The form and content of the system, etc., guide medical staff to form the concept of friendly communication from the intrinsic value orientation. At the same time, the communication skills of medical staff need to be improved. When communicating with patients, medical staff should pay attention to empathy, respect, and patience, maintain a good habit of listening, and communicate with patients after fully grasping the patient’s condition, treatment and examination results, and medical expenses. It is also necessary to pay attention to the patient’s emotional response to the disease treatment and avoid forcing the patient to accept the fact of the disease, avoid stimulating the other party’s emotions, or excessively exploiting the challenges faced by the other party when doctors use professional vocabulary for communication.

### 5.4. Narrowing the Knowledge and Information Gap and Enhancing the Equal Status of Information

The knowledge gap and information asymmetry between doctors and patients are unavoidable. However, it is possible to change. Due to the absolute advantage of medical knowledge and information, it is necessary to focus on patients to reduce the knowledge and information asymmetry between doctors and patients. Therefore, the channels for obtaining medical information can be broadened for patients to increase their understanding of their diseases, such as other authoritative information channels to understand the development history of the disease, the current status of diagnosis and treatment, and treatment methods to improve their medical quality. For the government, it can cooperate with the media to promote national health education and popularize medical knowledge; for example, in the community, by holding free lectures, distributing publicity materials, placing information display boards, playing publicity videos, etc., conducting health knowledge lectures on common diseases, and conduct health knowledge lectures. For the current topics of concern or diseases that need vigilance, targeted publicity and education programs are formulated, and relevant special editions or feature films are produced to popularize health knowledge.

### 5.5. Build an Error Prevention Mechanism and Improve the Error Early Warning Mechanism

Establishing a medical error prevention mechanism is one of the important ways to prevent medical errors. Improving medical technology improves communication, and training platforms should be established to avoid unnecessary diagnosis and treatment errors. For example, hospitals can conduct training and exchange activities on medical technology skills on a regular and irregular basis to improve the medical skills and level of medical staff. On the other hand, the hospital should give full play to the role and function of the medical quality committee, supervise, control, evaluate and punish errors in the hospital, ensure the safety of patients, and exhort medical staff to summarize experience and lessons promptly to avoid secondary errors. Finally, the information early warning system should be improved, the safe use of drugs and medical equipment should be automatically prompted, and early warning through modern information technology and the role of information technology in improving and preventing the quality of medical care should be brought into play. For example, by developing a scientific and practical prescription review system, an evidence-based medical system plays the role of automatic error warning.

## 6. Conclusions

Doctor–patient conflicts and disputes should be paid constant attention. The internal causes of doctor-patient disputes mainly include the government, hospitals, doctors, and patients. The lack of government supervision, inadequate hospital management, insufficient medical resources, improper behavior of doctors and nurses, faults of doctors in diagnosis and treatment, and subjective dissatisfaction of patients are important factors leading to doctor-patient disputes.

## 7. Limitation

This study is a qualitative analysis, and 622 case texts from the China Judgment Documents Network were selected for analysis. The sample has certain limitations. Later studies could include more case texts as well as actual cases. At the same time, there was little information about the doctors and patients involved in each case, and there was a lack of analysis of doctors’ data. Through interviews, subsequent research can explore the correlation between doctors’ personal characteristics and medical errors. In addition, subsequent research can also use quantitative methods and analyze the correlation between medical malpractice and doctors and hospitals.

## Figures and Tables

**Table 1:** Summary of doctor–patient disputes data in various provinces in 2020.

District	Quantity	Number of Public Hospital Cases	Sample Quantity	District	Quantity	Number of Public Hospital Cases	Sample Quantity
Jiangsu	1871	1665	75	Heilongjiang	372	331	15
Shandong	1634	1454	66	Fujian	366	326	15
Henan	1507	1341	61	Guangxi	363	323	15
Beijing	1080	961	43	Shanxi	360	320	14
Jilin	807	718	33	Shaanxi	252	224	10
Sichuan	746	664	30	Guizhou	251	223	10
Hebei	672	598	27	Shanghai	246	219	10
Hubei	615	547	25	Inner Mongolia	241	214	10
Zhejiang	524	466	21	Xinjiang	138	123	6
Hunan	499	444	20	Tianjin	134	119	5
Anhui	493	439	20	Ningxia	46	41	5
Liaoning	396	352	16	Gansu	28	25	3
Yunnan	394	351	16	Hainan	8	7	2
Guangdong	391	348	16	Qinghai	6	5	2
Chongqing	387	344	16	Tibet	1	1	1
Jiangxi	382	340	15				

**Table 2:** Open coding summary table.

Generic	Original Statement
Delay in diagnosis and treatment 288/622 (46.30%)	The defendant was at fault during the treatment process, and the rescue was not timely, causing the plaintiff to be disabled. The doctor did not treat in a time when the patient’s fetus was in distress. Before the cesarean section, all the tests were not timely. There are delays in the hospital’s diagnosis and treatment of patients with craniocerebral trauma, and the surgical treatment is not timely.
Missed diagnosis and misdiagnosed disease 52/622 (8.36%)	The doctor blindly concluded that the diagnosis is: asthma, chronic bronchitis, which is a misdiagnosis that violates common sense. Diagnosed as acute pharyngitis in the case of abnormal test results, used antibiotics for treatment. The hospital’s misdiagnosis and hysterectomy caused the plaintiff’s cervical cancer. A misdiagnosis of delayed encephalopathy caused by carbon monoxide poisoning delayed the best treatment period, and the doctor had apparent faults in the diagnosis and treatment process.
Improper use of drugs 73/622 (11.74%)	A drug skin test was not given to the patient before intravenous administration, resulting in drug anaphylactic shock and death. The medical practice of overdose in the use of antipyretics by the doctor; the overdose of the drug aggravated the inflammatory response of the child’s disease and aggravated the condition. Due to the doctor’s wrong prescribing of medication this directly led to the complete damage of the left facial nerve and the hypofunction of the bilevel semicircular canal.
Insufficient diagnostic examination 41/622 (6.59%)	Without taking any ECG monitoring and perfecting relevant examinations, the doctor blindly concluded and diagnosed asthma and chronic bronchitis. In the process of diagnosis and treatment, the hospital failed to perform the required inspection to rule out the possible risk of disease. The hospital did not check the patient’s right eye injury in place, underestimated the severity of the patient’s condition, and delayed the diagnosis and treatment of the disease.
Improper nursing operation 41/622 (6.59%)	Postoperative medication and nursing violated the norms of diagnosis, treatment, and nursing, and there were significant mistakes. When the nurse on duty was nursing the patient, she accidentally stuck tape on her glove and directly took the intubation tube out of the patient’s mouth. The nursing operation violated the “Clinical Nursing Practice Guidelines (2011 Edition)”.
Improper surgical operation 145/622 (23.31%)	There are problems such as non-compliance with the regulations in the doctor’s operation records. The doctor did not grasp the surgical indications rigorously and performed improper operations during the operation. The hospital’s operation was not rigorous enough, and the patient was required to be transferred to other hospitals for treatment after the operation.
Insufficient communication between doctors and patients 62/622 (9.97%)	Insufficient communication between doctors and patients on the significance of cerebral angiography examination, insufficient communication between doctors and patients. Insufficient assessment and treatment of the severity of the children’s condition, insufficient communication between doctors and patients. The doctor arranged for the patient to have an ECT examination at an inappropriate time, and there was no communication record between the doctor and the patient
Lack of professionalism 16/622 (2.57%)	The doctor concealed the patient’s condition and did not inform the patient’s actual condition to their guardian in time. The doctor was highly irresponsible and lost the test sheet, resulting in severe hypoxia after birth. The doctor was irresponsible, yelling at the patient and ignoring the patient’s pain.
Insufficient risk notification 67/622 (10.77%)	The doctor did not explain the medical alternatives to the patient, nor did he inform the pros and cons of the plan. Failure to inform patients and their families about the risk of brain damage during surgery. Failure to inform patients of postoperative risks and preventive measures after discharge.
Violation of medical record writing 161/622 (25.88%)	Failing to make, write and save outpatient medical records. The writing of medical records was not standardized, and there were apparent differences between the medical records and the actual situation. The outpatient medical records attached to the hematology department upon admission were handwritten and did not meet the norms.
Inferior equipment and consumables 36/622 (5.79%)	The use of unqualified internal fixation products by the doctor directly caused the bone destruction of the patient’s proximal right clavicle and sternal manubrium. The chemical composition of the same batch of broken steel nails did not meet the requirements of national standards, and the microstructure and hardness did not meet the requirements of relevant standards, resulting in the final breakage of steel nails. The disposable sterile package expired, causing infection incidents.
Practicing medicine beyond the scope 26/622 (4.18%)	The scope of practice of the treating physician did not meet the requirements. Doctors who examine, diagnose and operate on patients do not have a license to practice. The doctor does not have the qualifications to perform tertiary surgery, and the medical staff do not have the qualifications.
Lack of trust in diagnosis and treatment 88/622 (14.15%)	A patient believed that the doctor’s delivery method was improper and directly led to the death of their newborn. The patient provided the precautions collected on the Internet to prove the doctor’s mistake, but the evidence belongs to the network knowledge, and there is no corresponding factual and legal basis for whether it meets the medical regulations. During the hospitalization period, privately contacted acquaintances for barium dialysis of the digestive tract, which aggravated the condition of the patient.
Failure to cooperate with diagnosis and treatment 36/622 (5.79%)	The doctor suggested deep vein catheterization for parenteral nutrition, but the patient was hesitant and did not cooperate with the treatment in time. The patient was sent for rescue treatment in the ICU, because the relatives of the patient refused to cooperate with the treatment. The doctor advised her to give birth by cesarean section, but the puerpera and her family insisted on continuing vaginal trial delivery, which could lead to severe consequences such as obstructive dystocia, uterine rupture, vesicovaginal fistula, neonatal intracranial hemorrhage, and intrauterine death.
Disrupting the order of diagnosis and treatment 16/622 (2.57%)	During the doctor’s examination, the patient thought the doctor’s examination caused his child’s constant crying, so he maliciously pushed and shoved and caused the two parties to fight each other. Because the doctor was silent during the diagnosis and treatment, the patient thought that his attitude was horrible, and the doctor only cared about prescribing medicine to make money, so he violently attacked the doctor. Because the patient waited a long time for a prenatal checkup, she believed that the hospital service was not appropriate, so she insulted and beat the nurse on duty.
Dissatisfied with medical expenses 10/622 (1.61%)	Expend hundreds of thousands of yuan in medical expenses, unable to pay for the cost of surgery to remove the steel plate. In addition to the medical expenses incurred by normal childbirth, the remaining expenses are unreasonable or too high. The patient claims that the fee is unreasonable, and the subject of the claim should be the social security institution.
The patient’s condition is poor 57/622 (9.16%)	Elderly patients, who also have a variety of underlying diseases in the past and have immune dysfunction. The patient is older and has suffered from asthma for 30 years, and the threshold for resisting the influence of various internal or external factors on the body is lower than that of ordinary people. The diagnosis and treatment process is correct, and the surgical measures taken are appropriate. During the operation, due to laparoscopic surgery, the operation is minimally invasive, but the technology required is relatively high, and the patient’s disease is the reason for ureteral injury.
Resource and technical limitations 21/622 (3.38%)	It is a rare disease that is a medical problem, and the medical technology level of the county-level medical institutions could not diagnose the genetic disease in the fetal stage at that time. Factors such as local medical technology conditions. There is no necessary rescue equipment and no rescue technology, resulting in the death of the patient in the hospital due to lack of proper treatment after suffocation.

**Table 3:** Summary of main axis coding.

Main Generic	Sub-Generic	Number of Cases	Interpretation
Medical care diagnosis and treatment fault	Delay in diagnosis and treatment	228	Disputes caused by doctors’ mistakes in the process of diagnosis and treatment resulting in physical or psychological losses to patients
	Misdiagnosed and misdiagnosed	52	
	Improper use of drugs	73	
	Failed to perfect inspection	41	
	Improper nursing practice	41	
	Improper operation	145	
Lack of medical literacy	Insufficient doctor–patient communication	62	Conflicts caused by patients’ dissatisfaction with medical treatment due to insufficient service awareness and service ability of medical staff
	lack of professionalism	16	
Lack of hospital management	Insufficient risk communication	67	Conflicts caused by patients’ dissatisfaction with medical treatment due to inadequate implementation of relevant hospital management measures
	Medical record writing violations	161	
Lack of government regulation	Inferior equipment consumables	36	Due to the lack of government supervision, the illegal behavior of the hospital is not regulated, which leads to the harm of patients’ medical treatment
	Practicing medicine beyond the scope	26	
Patient dissatisfaction	Lack of trust in diagnosis and treatment	88	Conflicts with doctors due to differences in patients’ cognition of diseases and partial dissatisfaction with medical treatment
	Not cooperating with medical treatment	36	
	disrupt the medical order	16	
	Dissatisfied with medical expenses	10	
objective factors	The patient’s condition is poor	57	Refers to the contradictions and conflicts caused by objective factors that exist and are difficult to change
	Resource Technology Constraints	21	

**Table 4:** Typical relational structure of main categories.

Typical Relational Structure	The Connotation of Relational Structure	Theoretical Theme
Lack of government supervision → lack of hospital management → doctor–patient disputes	The lack of supervision of hospitals by competent government departments has led to the failure to detect and stop the illegal activities of some medical institutions in a timely manner. For example, hospitals use substandard medical consumables and equipment, and carry out related diagnosis and treatment activities beyond their scope of practice. Suffering from conflict and burying hidden dangers	Controlling Prisoners Affects Policy Implementation
Insufficient hospital management → Improper behavior of doctors and nurses → Disputes between doctors and patients	As a relatively special service organization, medical care has the responsibility and obligation to strengthen the service level and service awareness of medical staff in the organization, and regulate the behavior of medical staff, otherwise it will inevitably lead to “customers” dissatisfaction with the service.	
Insufficient hospital management → doctor’s fault in diagnosis and treatment → doctor–patient dispute	As the main place of medical activities, hospitals have the responsibility and obligation to manage and standardize the diagnosis and treatment activities of the hospital and require medical staff to standardize the performance of diagnosis and treatment procedures. doctor–patient conflict	
Doctor’s fault in diagnosis and treatment → doctor–patient dispute	In the diagnosis and treatment activities, the doctor’s fault diagnosis and treatment behavior caused by misdiagnosis; misdiagnosi and improper clinical operation directly affects the physical and mental health of patients and the treatment of diseases, and is the primary factor that induces doctor–patient conflict.	Damage to rights will inevitably lead to conflicts
Improper medical behavior → doctor–patient disputes	As medical service personnel, medical staff should have a good sense of service for patients. When receiving patients, the lack of service level and service awareness will lead to patients’ dissatisfaction with medical treatment, which will lead to conflicts.	
Subjective dissatisfaction of patients → doctor–patient disputes	Due to the patient’s cognitive asymmetry between the disease itself and the disease diagnosis and treatment information, the understanding of the disease treatment is biased, the failure to cooperate with the diagnosis and treatment or the wrong understanding of some diagnosis and treatment behaviors, eventually lead to conflicts and dissatisfaction between the doctor and the patient.	Information asymmetry leads to misunderstanding between doctors and patients
Restricted by objective factors → doctor’s fault in diagnosis and treatment → doctor–patient dispute	Due to the poor health of the patient, or the limitation of current medical technology, equipment, talents and other resources, this can lead to unavoidable non-fault diagnosis and treatment errors, which lead to contradictions and conflicts between doctors and patients.	The dilemma of technology and resources will inevitably lead to conflicts

## Data Availability

The data sets used and/or analyzed during the current study are available from the corresponding author on reasonable request.

## References

[1] Du L., Xu J., Chen X. (2020). Rebuild doctor–patient trust in medical service delivery in China. Sci. Rep..

[2] Wang F., Song Z., Zhang W., Xiao Y. (2017). Medical Humanities play an important role in improving the doctor-patient relationship. BioSci. Trends.

[3] Qi X.X. (2020). Cause Analysis and Legal Response to the "Intensification" of Doctor-Patient Disputes—Taking Three Cases of Violent Attacks on Doctors as Concerns. Qiushi Acad. J..

[4] Xu B. (2022). The impact of covid-19 on the doctor-patient relationship in China. Front. Public Health.

[5] Chang P.C., Wu T., Du J. (2020). Psychological contract violation and patient’s antisocial behaviour. Int. J. Confl. Manag..

[6] Liang J.L. (2010). Development of Western Health Sociology Research. Foreign Soc. Sci..

[7] Schauer A.J. (2001). Ethics in Medicine.

[8] Robinson G. (1989). A thought-provoking exchange. Nurs. Manag..

[9] Allman R.L. (2003). The relationship between physicians and the pharmaceutical industry: Ethical problems with the every-day conflict of interest. HEC Forum.

[10] Yang T.W., Lu W.T. (2006). A review of the research on conflict between doctors and patients at home and abroad. Chin. Med. Ethics.

[11] Shao H., Hu X.J. (2017). On the Refined Path of Doctor-Patient Dispute Prevention. Jinyang Acad. J..

[12] Wu L.H., Meng F.Y. (2020). Using Marxist philosophy to analyze the doctor-patient relationship and solutions in the new era. Contin. Med. Educ..

[13] He W., Wang X., Zhou X., Xu L. (2019). Negative expectations and bad relationships: Effects of negative metastereotypes on doctor–patient relationships. Asian J. Soc. Psychol..

[14] Gerds T.A., Kattan M.W. (2021). Medical Risk Prediction.

[15] Lerner I., Veil R., Nguyen D.P., Luu V.P., Jantzen R. (2018). Revolution in health care: How will data science impact doctor–patient relationships?. Front. Public Health.

[16] Rubin G.L., Frommer M.S. (2001). Evidence-based medicine—Time for a reality check. Med. J. Aust..

[17] Zhang X., Ma L., Ma Y., Yang X. (2021). Mobile information systems usage and doctor-patient relationships: An empirical study in China. Mob. Inf. Syst..

[18] Hu Y., Zhou H., Chen Y., Yao J., Su J. (2021). The influence of patient-generated reviews and doctor-patient relationship on online consultations in China. Electron. Commer. Res..

[19] Tao S., Liu C., Wu Q. (2021). Developing a scale measuring the doctor–patient relationship in China from the perspective of doctors. Fam. Pract..

[20] Ren W., Sun L., Tarimo C.S., Li Q., Wu J. (2021). The situation and influencing factors of outpatient satisfaction in large hospitals: Evidence from Henan Province, China. BMC Health Serv. Res..

[21] Zeng W., Ma S., Callan V.J., Wu L. (2021). Exploring the doctor-patient relationship as a challenge job demand: Application of the job demands–resources model in a chinese public hospital. Psychol. Health Med..

[22] Woolf N.H., Silver C. (2017). The architecture of NVivo. Qual. Anal. Using NVivo.

[23] Woolf N.H., Silver C. (2017). Orientation to nvivo. Qual. Anal. Using NVivo.

[24] Kendall J. (1999). Axial coding and the grounded theory controversy. West. J. Nurs. Res..

[25] Strübing J. (2014). Grounded theory und situationsanalyse: Zur Weiterentwicklung der grounded theory. Grounded Theory.

[26] Zhang L. (2021). On the construction of the characteristic indicators of effective education and scientific research in primary and secondary schools—Based on the NVivo coding analysis of 54 texts. Curric. Textb. Teach Methods.

[27] Mason M. (2010). Sample Size and Saturation in PhD Studies Using Qualitative Interviews. Forum Qual. Soz./Forum Qual. Soc. Res..

[28] Lai Y., Chen S., Li M., Ung C.O., Hu H. (2021). Policy interventions, development trends, and service innovations of internet hospitals in China: Documentary analysis and qualitative interview study. J. Med. Internet Res..

[29] Xu C., Luo L., Zeng S., He X., Li J., Zhu G. (2022). What promotes medical overuse: Perspective on evolutionary game between administration and Medical Institutions. Comput. Math. Methods Med..

[30] Cui C., Zuo X., Wang Y., Song H., Shi J., Meng K. (2020). A comparative study of patients’ satisfaction with different levels of hospitals in Beijing: Why do patients prefer high-level hospitals?. BMC Health Serv. Res..

[31] Lawton A.J., Rosenberg L.B. (2022). Carpe communication: Seizing the small moments to teach interpersonal and communication skills on inpatient services. J. Grad. Med. Educ..

[32] Liu J., Yu C., Li C., Han J. (2020). Cooperation or conflict in doctor-patient relationship? an analysis from the perspective of evolutionary game. IEEE Access.

[33] Brüggemann A.J., Wijma B., Swahnberg K. (2011). Abuse in health care: A concept analysis. Scand. J. Caring Sci..

[34] Rongjuan W. (2022). How multiple interactions between policy instruments and the policy environment affect environmental governance efficiency. Energy Environ..

[35] Butalid L., Verhaak P.F., Boeije H.R., Bensing J.M. (2012). Patients’ views on changes in doctor-patient communication between 1982 and 2001: A mixed-methods study. BMC Fam. Pract..

[36] Scheppokat K.D. (2005). Lehren aus den Erfahrungen einer schlichtungsstelle für arzthaftpflichtfragen. Ther. Umsch..

[37] Jiang F., Zhou H., Hu L. (2019). Psychiatry residents in China: Socio-demographic characteristics, career satisfaction, and related factors. Front. Psychiatry.

